# Effect of a Motion Artifact Correction System on Cone-Beam Computed Tomography Image Characteristics

**DOI:** 10.7759/cureus.35016

**Published:** 2023-02-15

**Authors:** Mayank Pahadia, Rujuta Katkar, Hassem Geha

**Affiliations:** 1 Oral and Maxillofacial Diagnostic Sciences, University of Florida, Gainesville, USA; 2 Comprehensive Dentistry/Oral and Maxillofacial Radiology, University of Texas Health Science Center at San Antonio, San Antonio, USA; 3 Oral and Maxillofacial Radiology, University of Texas Health Science Center at San Antonio, San Antonio, USA

**Keywords:** contrast resolution, ct artifacts, motion correction, maxillofacial radiology, cone-beam computed tomography (cbct)

## Abstract

Objectives: Determine the effect of the motion correction system on cone-beam computed tomography (CBCT) image quality parameters, artifacts, and contrast-to-noise ratio (CNR) using different motion settings.

Materials and methods: A customized phantom insert array was prepared using SEDENTEX CT IQ Phantom (Leeds Test Objects, Yorkshire, England) stabilized over a rotating electric turntable. Thirty baseline CBCT scans were acquired with standardized technique factors on the ProMax 3D (Planmeca, Helsinki, Finland) machine using combinations of different motion settings, including no motion, three- and six-degree motion, and with and without the use of a motion correction system. The standardized images were exported to ImageJ software. Image quality parameters, artifacts, and CNR values were evaluated and compared among the different acquisition settings.

Results: The use of the motion correction system algorithm compared with the different motion settings showed a statistically significant difference for all the parameters (p<0.05) except for artifact values for six-degree motion (p<0.07). The effect of different motion settings on the parameters was not statistically significant.

Conclusion: The use of a motion correction system, a proprietary algorithm-based system incorporated in the ProMax 3D CBCT unit, deteriorates the image quality characteristics evaluated in this in vitro study, namely artifact value and CNR. Its use in clinical settings might be limited to situations where patient motion is expected and appropriate head stabilization is not possible due to age or disease.

## Introduction

Over the last decade, the use of cone-beam computed tomography (CBCT) for various diagnostic tasks in oral and maxillofacial imaging across the world has exponentially increased. It has become a standard for imaging for dental implant assessment, endodontic evaluation, various jaw lesions, etc. From a technical point of view, to produce radiographic images, a divergent pyramidal or cone-shaped source of ionizing radiation is directed through the area of interest onto an area on the X-ray detector that is present on the opposite side. The X-ray source and detector rotate around a rotation fulcrum fixed within the center of the region of interest. During the rotation, multiple (from 150 to more than 600) sequential planar projection images of the field of view (FOV) are acquired in a complete, or sometimes partial, arc. Once the basis projection frames have been acquired, data must be processed to create the volumetric data set. This process is called reconstruction. Reconstruction is a multistep process that is computationally complex. The most common reconstruction method is the Feldkamp algorithm, which is a modified filtered back projection method [1].

Despite the advantages that CBCT imaging provides, there are several limitations. Limited soft-tissue contrast resolution and the presence of multiple artifacts in the final reconstructed images are significant limitations. In CBCT imaging, an artifact may be defined as a "visualized structure in the reconstructed data that is not present in the object under investigation." Generally, artifacts are introduced by discrepancies between the actual physical conditions of the CBCT scanner's technical composition and the composition, position, and behavior of the object under consideration. Another reason is the simplified mathematical assumptions used for 3D image reconstruction [2].

Among the artifacts that arise in CBCT, artifacts due to patient motion are prevalent, yet they are relatively unexplored. The general problem is quite easy to explain in terms of patient motion artifacts. Suppose an object moves during the scanning process. The reconstruction does not account for that movement since no information on the motion is integrated into the reconstruction process. When the object moves, the back-projection lines do not correspond to the lines where the attenuation is recorded. The back projection assumes a completely stationary geometry. Consequently, the intensities contained in the projections are back-projected under the static assumption. A correction compensating the actual movement effective in each of the projections would be required [2].

The relatively long acquisition times for some CBCT scans, ranging from 5 to 40 seconds, make it difficult for patients' ability to keep still during the examination, particularly children. Further, other reasons for patient movements, such as systemic diseases in elderly patients (e.g., Parkinson's disease), must be considered. If the artifacts are severe, leading to a significant loss in image quality, the CBCT image sections may not be interpretable, and scans may need to be acquired again, thereby increasing the total patient dose [3].

Minimal patient movement can cause motion artifacts, potentially degrading image quality. Recent in vivo studies showed that movements ≥0.5 mm take place in nearly 80% of CBCT examinations [4], and suggested that the presence of patient movement ≥3 mm had a significant impact on CBCT image quality and interpretability [5]. To overcome the drawbacks of patient motion, the literature has suggested that CBCT reconstruction algorithms should consider patient motion, i.e., provide automated correction of motion artifacts [3].

To the best of our knowledge, only two CBCT scanners, ProMax 3D (Planmeca, Helsinki, Finland) and X1 (3Shape, Denmark), have incorporated motion artifact correction systems to date. The X1 correction system requires a head tracking system, without which motion artifact correction cannot happen. However, the ProMax 3D uses a correction algorithm that does not require a head-tracking device and can be turned on and off before scan acquisition [6]. The motion artifact correction system in ProMax 3D is called CALMTM (Correction Algorithm for Latent Movement). In this in vitro study, we evaluated the effect of a motion correction system on CBCT image quality parameters, namely artifacts and contrast-to-noise ratio (CNR), using different motion settings.

## Materials and methods

Thirty baseline CBCT scans were acquired using the ProMax 3D (Planmeca, Helsinki, Finland) unit with standardized acquisition parameters: 10 cm × 10 cm field of view (FOV), 90 kVp, 12 mA, and 15 seconds exposure time. The voxel size selected was 150 µm.

**Figure 1:**
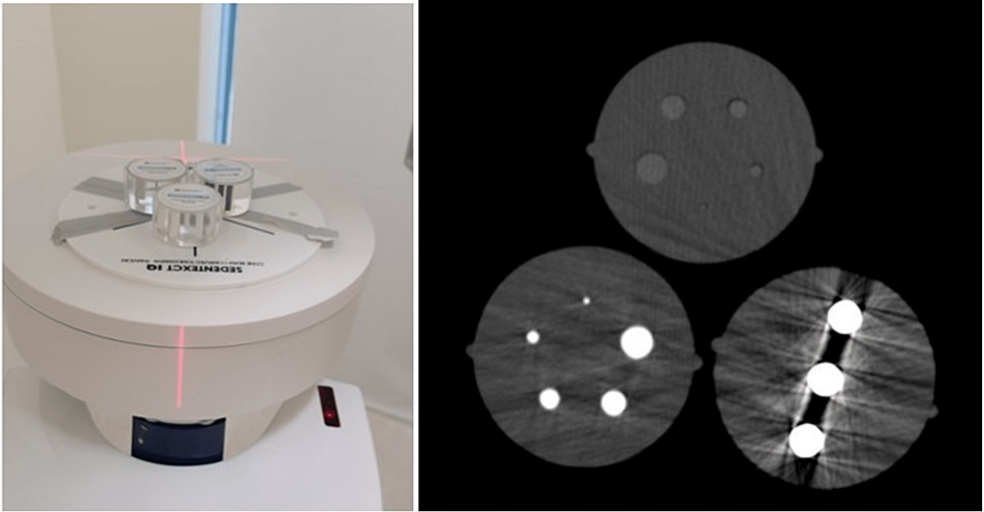
(a) Placement of custom array of SEDENTEX CT IQ Phantom inserts stabilized over rotating electric turntable; (b) axial CBCT image post-acquisition of the phantom inserts

The SEDENTEXCT IQ Phantom, developed by Leeds Test Objects Ltd. (Boroughbridge, North Yorkshire, England), was used for image quality testing [7]. A customized phantom array with three inserts was prepared. The inserts for artifacts, the contrast resolution for aluminum, and the contrast resolution for delrin were used. The inserts were positioned such that they would be visible and centered in the images (Figure 1a-1b).

A motorized electric turntable (JAYEGT, Shenzhen Kayak Technology Co., Ltd., China) with 360-degree rotation and a speed of 12 revolutions per second was used to induce the motion for image acquisition. The device allowed for remote-controlled rotation at various degrees, as low as 1-degree to-and-fro motion. The phantom array was placed, centered, and stabilized over the rotating turntable for CBCT image acquisition (Figure 1a-1b).

Six different scan combinations were acquired with the use of the CALM™ algorithm and degree of motion. The motions used with the combinations are given in Table 1.

**Table 1:** Six scan combinations with variations in motion and use of motion correction system

Number	Scan combination
1	Without motion without motion correction system
2	Without motion with motion correction system
3	Three-degree motion without motion correction system
4	Three-degree motion with motion correction system
5	Six-degree motion without motion correction system
6	Six-degree motion with motion correction system

The motion was induced after scan acquisition had begun, as soon as the gantry rotation was visually noted. For each combination, five baseline scans at the same acquisition setting were acquired to standardize the analysis. Scout images were acquired to verify the position of the inserts in the scan before the final acquisition. All thirty scans were acquired on the same day to prevent any change in the rotating turntable or phantom array position.

As per the SEDENTEX CT IQ Phantom user manual, native axial images were exported using the thinnest slice thickness and smallest slice interval available [7]. The images were exported into ImageJ software (National Institute of Health, Bethesda, MD, USA) for analysis of artifacts, contrast resolution, and CNR. The analyses were as follows.

For artifact (via beam hardening artifact)

**Figure 2:**
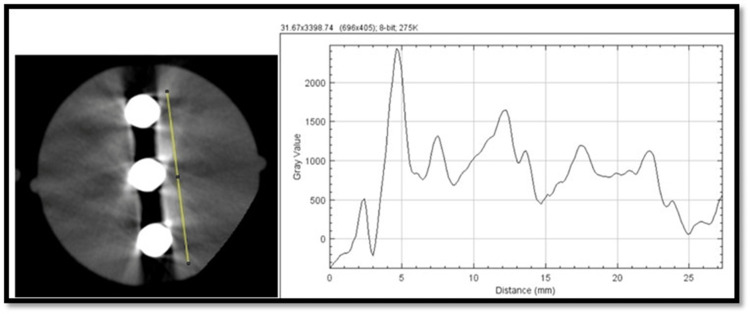
Axial CBCT image showing artifact insert and plot profile generated through ImageJ software indicating gray values for analysis

We selected the 100th slice from the scan's inferior aspect, which provided optimum visualization of the phantom inserts. The insert consists of a line of three 5.0 mm diameter rods of titanium suspended in a polymethyl methacrylate (PMMA) base [7]. A 25-mm line connecting the peripheral edges of the insert that was parallel and 2 mm away from the three rods was drawn and exported to ImageJ for plot profile analysis. A macro was created and used to maintain reproducibility. The peaks and valleys on the resulting gray value plot were examined, and maximum and minimum values were noted. The difference between these maximum and minimum values was defined as the artifact (Figure 2).

For contrast-to-noise ratio

**Figure 3:**
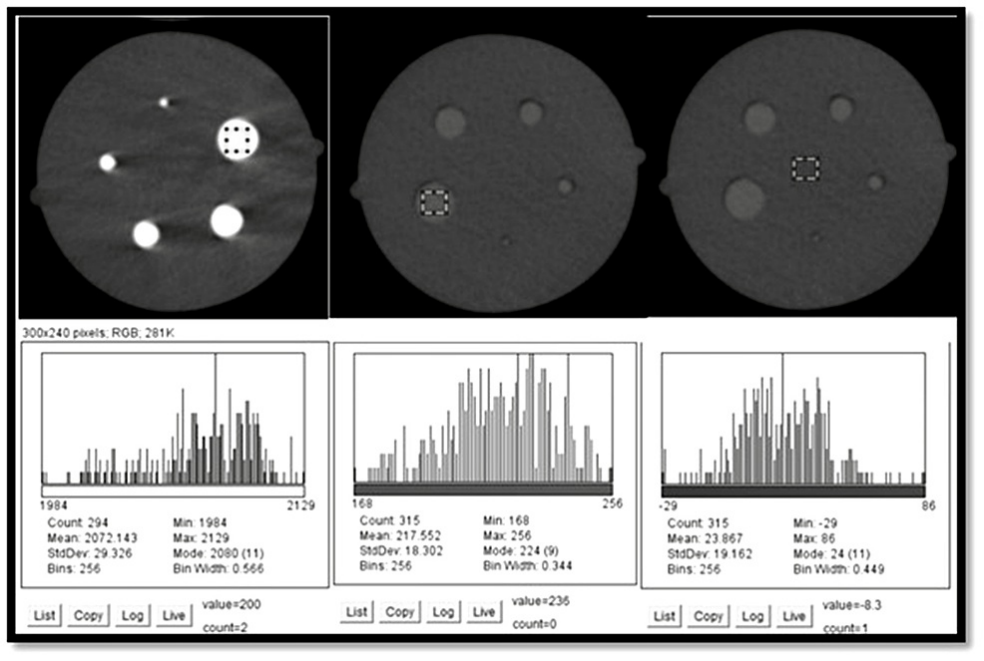
Area analysis for contrast resolution and CNR for aluminum, delrin and control area with representative histograms with mean and standard deviation values

We selected the 100th slice from the scan's inferior aspect, which provided optimum visualization of the phantom inserts. Two types of contrast-resolution inserts were used: aluminum and delrin. Each insert consists of 5.0, 4.0, 3.0, 2.0, and 1.0 mm-diameter rods suspended in a PMMA base [7]. For CNR evaluation, the two contrast resolution inserts and a control area in the PMMA base were selected. A 4 mm × 4 mm square centered area in the 5.0 mm diameter rods was selected in the aluminum contrast resolution insert, and a histogram was created using ImageJ. Similar to artifact evaluation, a macro was created for reproducibility. The mean and standard deviation values were recorded from the histogram. Subsequent mean and standard deviation values were recorded for the Delrin insert and control area (Figure 3).

The CNR was then calculated for both aluminum insert and delrin inserts using the formula [8]:

CNR_Aluminum_ = Mean _Aluminum_ − Mean _Control_ / \(\sqrt{SD^{2}_Aluminum__{} + SD^{2}_Control__{_{}}}\)

CNR_Delrin _= Mean _Delrin_ − Mean _Control_ / \(\sqrt{SD^{2}_Delrin__{} + SD^{2}_Control__{_{}}}\)

The six scan combinations with five baseline scans per combination were evaluated for artifact and CNR values, and the mean values were used for each scan combination.

Statistical analysis

The statistical analysis was done using SPSS software version 23.0. A student t-test was used to evaluate the effect of the motion correction system on each motion setting separately. A one-way ANOVA was used to evaluate the three motion settings with and without the use of the motion correction system. All six setting combinations were compared with each other using the post-hoc Bonferroni test. For all statistical tests, a p-value of <0.05 was considered statistically significant.

## Results

For each motion and motion correction system setting combination, five scan benchmark mean values for the artifact, CNR_Aluminum_, and CNR_Delrin_ were calculated, as shown in Table 2.

**Table 2:** Descriptive values of artifact, CNR aluminum and CNR delrin

Motion	Motion correction system	Artifact		CNR_Aluminum_		CNR_Delrin_	
		Mean	± SD	Mean	± SD	Mean	± SD
Without motion	Without motion correction system	1204.20	59.19	59.52	4.81	7.77	0.75
	With motion correction system	1780.64	103.17	30.17	7.69	5.09	0.60
Three degree motion	Without motion correction system	1222.19	131.17	53.15	6.54	7.76	1.10
	With motion correction system	1804.60	97.12	28.76	9.59	5.30	0.49
Six degree motion	Without motion correction system	1498.44	351.52	49.49	7.10	6.96	0.57
	With motion correction system	1832.42	108.27	33.83	6.99	4.88	0.35

Effect of the use of the motion correction system for each motion setting (using a student t-test)

The use of the motion correction system for each motion setting separately was statistically significant (p<0.05) for the different values of artifact, CNR_Aluminum_, and CNR_Delrin_ except for the six-degree motion setting, where the artifact values were not statistically significant with or without the use of the motion correction system for scan acquisition (p>0.05) (Table 3).

**Table 3:** Comparing effect of motion correction system on each motion setting separately *Statistically significant.

Motion	Motion correction system	Artifact value			CNR_Aluminum_			CNR_Delrin_		
		Mean	± SD	P-value	Mean	± SD	P-value	Mean	± SD	P-value
Without motion	Without motion correction system	1204.20	59.19	<0.001*	59.52	4.81	<0.001*	7.77	0.75	<0.001*
	With motion correction system	1780.64	103.17		30.17	7.69		5.09	0.60	
3 degree motion	Without motion correction system	1222.19	131.17	<0.001*	53.15	6.54	0.002*	7.76	1.10	0.002*
	With motion correction system	1804.60	97.12		28.76	9.59		5.30	0.49	
6 degree motion	Without motion correction system	1498.44	351.52	0.077; NS	49.49	7.10	0.008*	6.96	0.573	<0.001*
	With motion correction system	1832.42	108.27		33.83	6.99		4.88	0.3	

Comparison of effect of each motion setting with and without the use of motion correction system (using One-way ANOVA)

The values of artifact, CNR_Aluminum_, and CNR_Delrin_ are not statistically significant for the different motion settings with and without the use of a motion correction system (p-values > 0.05) (Table 4).

**Table 4:** Comparing all three motion settings with and without use of motion correction algorithm ^#^One-way ANOVA.

Motion correction system	Motion	Artifact value			CNR_Aluminum_			CNR_Delrin_		
		Mean	±SD	P-value^#^	Mean	±SD	P-value^#^	Mean	±SD	Pvalue^#^
Without motion correction system	Without motion	1204.20	59.19	0.099; NS	59.52	4.81	0.071; NS	7.77	0.75	0.249; NS
	Three degree motion	1222.19	131.17		53.15	6.54		7.76	1.10	
	Six degree motion	1498.44	351.52		49.49	7.10		6.96	0.57	
With motion correction system	Without motion	1780.64	103.17	0.734; NS	30.17	7.69	0.612; NS	5.09	0.60	0.428; NS
	Three degree motion	1804.60	97.12		28.76	9.59		5.30	0.49	
	Six degree motion	1832.42	108.27		33.83	6.99		4.88	0.35	

Comparison of all six scan acquisition settings with each other (using post-hoc Bonferroni test)

The comparison of all scan acquisition settings with each other further emphasizes our results. There is a statistically significant difference in the use of the motion correction system compared with the different motion settings, except for the artifact values for the six-degree motion setting with and without using the motion correction system (Table 5).

**Table 5:** Comparison of all individual settings with each other *Statistically significant.

Comparison	Artifact value		CNR_Aluminum_		CNR_Delrin_	
	Mean difference	P-value	Mean difference	P-value	Mean difference	P-value
Without motion without motion correction system vs without motion with motion correction system	576.44	<0.001*	29.35	<0.001*	2.68	<0.001*
Without motion without motion correction system vs 3 degree motion without motion correction system	17.99	1.000; NS	6.379	1.000; NS	0.009	1.000; NS
Without motion without motion correction system vs 3 degree motion with motion correction system	600.39	<0.001*	30.76	<0.001*	2.47	<0.001*
Without motion without motion correction system vs 6 degree motion without motion correction system	294.24	0.181; NS	10.03	0.585; NS	0.81	1.000; NS
Without motion without motion correction system vs 6 degree motion with motion correction system	628.22	<0.001*	25.69	<0.001*	2.89	<0.001*
Without motion with motion correction system vs 3 degree motion without motion correction system	558.45	<0.001*	22.97	0.001*	2.67	<0.001*
Without motion with motion correction system vs 3 degree motion with motion correction system	23.95	1.000; NS	1.41	1.000; NS	0.20	1.000; NS
Without Motion with motion correction system vs 6 degree motion without motion correction system	282.20	0.233; NS	19.31	0.005*	1.86	0.004*
Without motion with motion correction system vs 6 degree motion with motion correction system	51.77	1.000; NS	3.65	1.000; NS	0.21	1.000; NS
3 degree motion without motion correction system vs 3 degree motion with motion correction system	582.40	<0.001*	24.28	<0.001*	2.46	<0.001*
3 degree motion without motion correction system vs 6 degree motion without motion correction system	276.24	0.264; NS	3.65	1.000; NS	0.80	1.000; NS
3 degree motion without motion correction system vs 6 degree motion with motion correction system	610.23	<0.001*	19.31	0.005*	2.88	<0.001*
3 degree motion with motion correction system vs 6 degree motion without motion correction system	306.15	0.140; NS	20.72	0.002*	1.65	0.013*
3 degree motion with motion correction system vs 6 degree motion with motion correction system	27.82	1.000; NS	5.06	1.000; NS	0.42	1.000; NS
6 degree motion without motion correction system vs 6 degree motion with motion correction system	333.98	0.076; NS	15.65	0.035*	2.08	0.001*

## Discussion

Although limited, several methods have been introduced for the detection, evaluation, and subsequent correction of patient motion in CBCT scans. Schulze et al. proposed an automated patient movement detection software based on optical flow theory, which is defined as the distribution of apparent velocities of movement of brightness patterns in an image. They concluded that optical flow theory is an efficient concept for the automated detection of patient motion on the projection images acquired during a CBCT scan [9].

Niebler et al. presented an iterative motion correction algorithm that allowed the patient's motion to be detected and taken into account during reconstruction. They observed improvements in image quality compared to uncorrected reconstructions, but the proposed algorithm is costly and computationally extensive [10].

Two CBCT manufacturers have incorporated motion correction systems into their machines, namely the X1 and ProMax 3D. Although their algorithms' exact functioning is not available due to proprietary restrictions, a basic understanding of how they function is available. X1 (3Shape, Denmark) requires a head tracking system for the detection of motion. The correction system is a two-step process. Step one is the tracking of the skull-robot movements using the head tracker apparatus integrated with the unit. The second step is an iterative reconstruction of the acquired projection images, incorporating the head tracker data [5]. The motion correction system CALM™ included in the ProMax 3D CBCT unit is understood to be an algorithm-based method, relying on optical flow measurements to predict movements as detected in the projection images. Due to the nature of the methods used to avoid motion artifacts, one could speculate that the latter somewhat compensates instead of correcting movement artifacts as optical flow measurements are directly related to brightness variation in the images. It may be affected by the inherent voxel value variation present in CBCT data sets, leading to inaccuracies in patient movement tracking [6].

Apart from our study, the effect of using the motion correction system has been shown in two studies. Spin Neto et al. compared the motion and motion-artifact-correction-system effects on apical periodontitis diagnosis in a human cadaver. They induced three motion types (3 mm movement with nodding, lateral rotation, and tremor type). They found that motion and motion correction systems had a significant effect on apical periodontitis diagnosis on CBCT images. The area under the curve (AUC) for images with the motion correction algorithm was 0.732-0.790, which was higher than the images without motion correction (AUC 0.541-0.70) [11].

In our study, the magnitude of the motion was slightly different. We induced motion using the electric turntable and increased the degree of motion while keeping the direction of the motion constant. In the study by Spin Neto et al. mentioned above [11], they changed the motion direction with different movement types while keeping the same 3 mm motion.

Santaella et al. compared the effects of different motion and motion artifact correction systems on image quality and interpretation using aligned and lateral-offset detectors. They used several diagnostic tasks, including implant planning and furcation assessment for interpretation, and six different types of motion, and compared the effect of these motions on the observers' interpretation abilities using different CBCT units, including ProMax 3D and X1, which had motion correction systems. They concluded that interpretation ability was improved with a motion correction system enabled for aligned detectors but was less effective with lateral off-set detectors [6]. Our study did not change the detector alignment for scan acquisition; movements were of three types based on the degree of motion rather than direction, and they only used a single motion correction system instead of two in their study.

Several other studies have been done, showing the effect of motion artifacts on images or different patient positioning on motion artifacts. Nardi et al. compared the effect of different head movements on image quality. They concluded that not all motion types affect the image quality equally, and movements of short duration and gradual movements affect the image differently [12]. Keris et al. did a retrospective study comparing the effect of motion on images with different patient positioning and found no significant impact of motion positioning [13]. However, while evaluating the effect of different types of motion on images, both of these studies are subjective and do not consider the possibility of motion correction systems.

To the best of our knowledge, this is the first study comparing the effect of the proprietary motion correction system CALM™ on the image quality characteristics, artifacts, and CNR values concerning different motion settings in an in-vitro setting using a CBCT phantom. It can be inferred from our study that, with the motion correction system enabled, there is a detrimental effect on the artifact values and CNR for both high and low-contrast objects. The image quality differences caused by the induction of motion at different levels were not statistically significant; however, they showed gradual image degradation.

Despite promising results, our study has several limitations. Although only one direction of motion was induced, its magnitude increased up to 6 degrees to and fro. In patients, especially in children, motion can occur in several directions, including anteroposterior or lateral; old patients with systemic diseases show tremor-like movements as well. Our study was performed in a controlled setting, and patient motion can affect different characteristics, including differences in scan acquisition times, differences in the age of the patient, and differences in projection geometry, including changes in the field of view of detector alignment. One of the important characteristics is spatial resolution, and although we incorporated it during the pilot project of this study, it was a visual assessment, and further studies are advocated using detailed modulated transfer function (MTF) calculations. With these limitations, further studies are advocated for evaluating these promising motion correction systems. Further studies should include spatial resolution and different types of motion and incorporate them into clinical applications. It should also be noted that the motion correction system can only be applied before scan acquisition, which limits its use in situations where excessive motion is expected to happen during scan acquisition.

Our results indicate judicious use of the motion correction system, as it can have a detrimental effect on the CNR and artifact values. Several factors affect patient motion during CBCT image acquisition, and the most important factors are scan acquisition time and patient head stabilization. Scan acquisition times can be reduced to a certain extent by reducing scan resolution, the number of basis images, or rotational arc, as permitted by the diagnostic needs of each scan. The second method is head stabilization, which can be performed using headrests, chin cups, or head restrainers. If optimum patient head stabilization and appropriate adjustment of scan acquisition times are possible, the motion correction system CALM™ needs to be carefully used as it may cause degradation of some image quality parameters.

## Conclusions

The use of the motion correction system, a proprietary algorithm-based system incorporated in the ProMax 3D CBCT unit, deteriorates the image quality characteristics evaluated in this in vitro study, namely artifact value and CNR. Its use in clinical settings might be limited to situations where patient motion is expected and appropriate head stabilization is not possible due to age or disease. However, there are other technical and clinical characteristics that need to be taken into consideration, and further studies are required.
